# Designing and psychometric assessment of the moral intelligence scale for healthcare professionals

**DOI:** 10.1038/s41598-024-55052-2

**Published:** 2024-02-24

**Authors:** Fateme Mohammadi, Seyed Reza Borzou, Salman Khazaei, Mostafa Bijani, Seyedeh Zahra Masoumi, Seyed Kianoosh Hosseini

**Affiliations:** 1 Department of Nursing, School of Nursing and Midwifery, Chronic Diseases (Home care) Research Center, Hamadan University of Medical Sciences, Hamadan, Iran; 2 Research Center for Health Sciences, Hamadan University of Medical Science, Hamadan, Iran; 3 Department of Medical-Surgical Nursing, Fasa University of Medical Sciences, Fasa, Iran; 4 Department of Midwifery, School of Nursing and Midwifery, Mother and Child Care Research Center, Hamadan University of Medical Sciences, Hamadan, Iran; 5 Department of Cardiology, School of Medicine, Farshchian Cardiovascular Subspecialty Medical Center, Hamadan University of Medical Sciences, Hamadan, Iran

**Keywords:** Competence, Moral, Health care, Professional, Cardiac, Operating room, Cardiology, Health care, Medical research

## Abstract

The moral intelligence of healthcare professionals in the cardiac operating room is one of the most important aspects of professional competence. However, moral intelligence is an abstract and multidimensional concept that needs to be clarified and described based on organizational culture and environment. Therefore, there is a need to design a specific scale for measuring the moral intelligence of healthcare professionals in the cardiac operating room. This study aims to design and assess the psychometric properties of a moral intelligence scale for healthcare professionals in the cardiac operating room. The present study was a mixed method study with a sequential exploratory approach. The research was conducted in 2023–2024 in Iran. The first phase data were collected from 20 healthcare professionals and were analyzed by conventional content analysis method. In the second phase, the validity and reliability of the instrument were evaluated by involving 300 healthcare professionals in the cardiac operating room. The moral intelligence of health care professionals in the cardiac operating room was defined as moral sensitivity combined with moral commitment and moral courage for the provision of quality care that respects the principles of medical ethics. After deducing the conceptual framework, the moral intelligence scale for healthcare professionals in the cardiac operating room was developed with three dimensions: “moral sensitivity,” “moral commitment,” and “moral courage.” 11 items were removed during testing to ensure content validity. Face validity was confirmed with impact scores > 1.5 for all items. A scale was developed through factor analysis with three factors that accounted for 73.04% of the observed variance. The instrument’s reliability using Cronbach’s alpha coefficient calculation was reported as 0.94 for the entire instrument. The testretest showed no statistically significant difference between the pre and post-test scores of moral intelligence (*p* = 0.51). The moral intelligence scale demonstrated acceptable psychometric properties. The moral intelligence scale for health care professionals in the cardiac operating room demonstrated acceptable psychometric properties. This instrument may serve to assess the moral intelligence of healthcare professionals and determine the need for educational interventions to reduce the ethical challenges and improve the moral intelligence of this healthcare.

## Introduction

Professional competence is one of the most important and challenging issues in healthcare, as one of the main missions of healthcare professionals is to provide moral care to individuals in society^1^. Therefore, acquiring and improving professional competence is crucial for healthcare professions^2^. Professional competencies are skills, knowledge and attributes that are specifically valued to your future career^1,2^. An important aspect of professional competence is adherence to principles and moral competence^3^. Moral competence in medical professions is defined as possessing moral knowledge, respecting individual and cultural differences, having the skills and abilities to deal with moral issues, and making the best and most ethical decision^4,5^. There are several moral theories, including moral foundations theory, moral judgment theory, moral decision-making theory, that having the skills and abilities to deal with moral issues and making the best moral decisions in health professions is in line with moral decision-making theory^4,5^.

Moral decision-making is a multifaceted process that requires individuals to make choices that can harm or benefit others consistently. It demands a delicate balance between personal and other interests, immediate or delayed gratification, and emotional and rational considerations^6,7^. Recent studies have revealed that moral decision-making is influenced by two primary computational processes^6,8^. The first concerns moral intuition, consisting of an emotional process that allows individuals to evaluate socially relevant stimuli as right or wrong; the second concerns moral reasoning, consisting of some controlled deductive reasoning processes and cost–benefit analyses about potential outcomes of moral decisions^6,8,9^. The ability to correctly recognize the situation and make suitable and proficient ethical decisions in these circumstances maybe is a testament to their professional competence, attitude and moral intelligence^6,10,11^. However, moral intelligence is the first component of ethical conformity and as the foundation of ethical practice guides that healthcare professionals in providing their patients with effective and ethical care^10,12^.A clear and precise definition of the concept of moral intelligence in healthcare professionals is not available. This is while it seems that this concept is influenced by education, beliefs, values, culture and society^10,11^.

The operating room is one of the most challenging and stressful therapeutic environments because patients enter the operating room without any companions and are exposed to a stressful environment with complex equipment while undergoing anesthesia and surgery and they have no support except health caregivers^13,14^. Respecting the four principles of medical ethics as described by Beauchamp and Childress (e.g., respect for patient autonomy, beneficence, non-maleficence, justice)^15^ is crucial for healthcare professionals as it plays a significant role for the provision of quality care^5^. Bilik et al. emphasize the importance of moral responsibility among healthcare professionals in the operating room. They suggest that healthcare policymakers and managers should develop appropriate programs to enhance their moral intelligence^16^. Additionally, Stievano et al. highlight the confusion among healthcare professionals in the operating room of various countries regarding their moral performance and decision-making, indicating a need for more precise and continuous training^17^.

However, heart disease affects approximately 500 million individuals globally, with an annual death toll of 18.6 million^18^. According to the Global Burden of Disease (GBD) study, cardiovascular diseases caused 285.33 (95%UI: 256.79–301.53) age-standardized mortality per 100,000 people in 2017 in Iran^19,20^. On the other hand, the National and Subnational Burden of Diseases, Injuries, and Risk Factors (NASBOD) study in 2015 showed that cardiovascular diseases caused 170.16 deaths per 100,000 people, accounting for 37% of all deaths in Iran^20,21^. Most of these patients undergo procedures such as percutaneous coronary intervention (PCI) and coronary artery bypass grafting (CABG)^22^. However, healthcare professionals working in cardiac operating rooms face demanding and stressful circumstances requiring clinical expertise, moral attitude and intelligence^23^.

Consequently, evaluating the moral intelligence of healthcare professionals is essential as a crucial aspect of professional competence^10^. The moral competence test is one of the most commonly used scales for measuring the ethical competency of health caregivers. This scale was designed by Lind in 2000 to evaluate moral competence. The scale presents three ethical dilemmas, with six arguments in favor and six arguments against each dilemma. Participants are asked to rate their arguments from completely disagree to completely agree^24,25^. Although this scale has been used in some studies to assess the moral competence of healthcare professionals, it was not originally developed for this purpose. Hence, it would be beneficial to create a more tailored scale that accurately evaluates the moral intelligence of healthcare professionals in each community’s cultural context. Additionally, Asahara et al. designed a moral competence assessment scale for home care nurses in Japan in 2013, consisting of 45 questions in five dimensions: moral sensitivity, moral judgment, moral motivation, moral personality, and moral decision-making. This scale was designed to focus on the moral competence of home care professionals^26^. Given that the treatment environment and patient needs in the operating room are different from those of in-home care, there is a need to design a more specific scale.

Due to the lack of clarity and multidimensionality of moral intelligence, it is impossible to provide a comprehensive definition of it^11^. Moreover, the moral intelligence of healthcare professionals is highly dependent on culture and society^10^. Despite numerous efforts to define this concept, identify influential factors, and design appropriate scales, the concept of moral intelligence remains complex and ambiguous. So, the purpose of the present study was to design and psychometrically evaluate the moral intelligence scale in healthcare professionals in the cardiac operating room in Iran.

## Methods

A mixed methods approach was used, employing a sequential exploratory design. In the initial phase, interviews were conducted with 20 healthcare professionals to identify and analyze key themes using conventional content analysis. The second phase involved developing and refining a 50-item questionnaire with input from a panel of 15 experts. This questionnaire was pretested with 30 healthcare professionals. Subsequently, a shorter 30-item questionnaire was created, utilizing a five-point Likert scale, and tested with a larger sample of 300 healthcare professionals. The psychometric properties of the questionnaire were assessed through factor analysis and reliability testing.

### Phase I. Instrument development

#### Qualitative study

In this phase of the present study, the researchers aimed to define and explain the concept of moral intelligence in the cardiac operating room from the perspective of healthcare professionals. To achieve this, they utilized the qualitative content analysis method. Because qualitative content analysis research can help explain a phenomenon in the cultural context of people’s perspectives who deal with a phenomenon for a long time^27^. Conventional content analysis is one of the most common and important qualitative content analysis methods. It allows for a better understanding of how individuals perceive and make sense of a phenomenon by identifying both commonalities and differences in their interpretations^28^. Also, conventional qualitative content analysis is an appropriate procedure for obtaining reliable and valid results from textual data, allowing the creation of new knowledge and innovative understanding of phenomena under investigation^29^. Therefore, a qualitative approach with conventional content analysis has been used to investigate this subject.

Twenty healthcare professionals from cardiac operating rooms in two public centers affiliated with medical universities in Iran’s west and southeast regions were selected with purposeful sampling. The criteria for inclusion were being willing to participate, having at least 12 months of work experience in the cardiac operating room, being Iranian, and having a good command of Farsi.

The study utilized various methods for data collection, including face-to-face, semi-structured interviews, observation, and field notes. A total of 20 healthcare professionals were interviewed in quiet hospital settings, when the participants did not have a shift. The time and location of the interviews were selected based on the participants’ preferences. Additionally, participants were asked to permit us to observe their performance for one working day during each interview. All participants agreed to allow us to observe their performance in the operating room for one working day. As a result, data for this study were collected through semi-structured interviews and observations of 20 professional healthcare workers.

**Figure 1:**
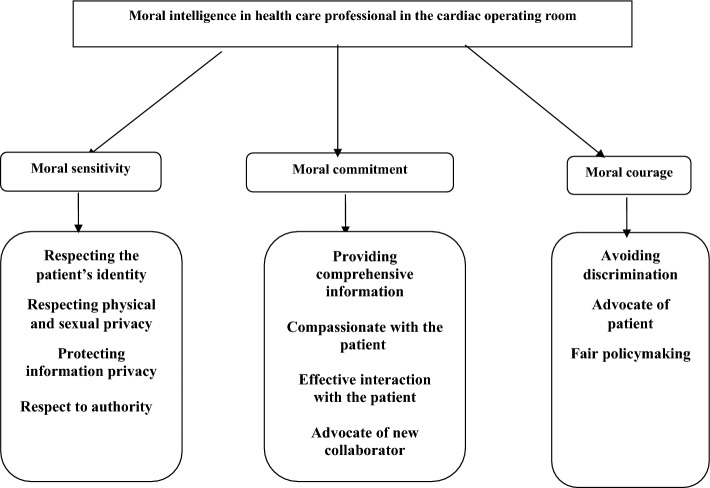
The main themes and subthemes in health care professional’s perceptions.

The interviews were thorough and aimed to understand the participants’ perspectives better. Each interview began with a few general questions, including” “Can you describe a day of your working in the cardiac operating room”? and” What do you think competence means for health care professionals in the cardiac operating room?”, “What factors affect the competence of the personnel in the cardiac operating room?” “What does moral intelligence mean to you?” “In your opinion, what factors are effective in the moral intelligence of personnel in the cardiac operating room?”. Subsequently, based on the ’respondents’ answers, follow-up questions would be asked to increase the clarity of the information—the questions included, “Can you explain further?”, “What do you mean by that?” and “Can you give an example?”. Based on the participant’s answers, other questions were asked to probe other aspects of moral intelligence further. The interviews were audio-recorded, observation and field notes were taken with the permission and awareness of the participants. Each interview lasted between 50 and 70 min. Immediately after conducting each interview, the first author listened to the recordings multiple times to gain a comprehensive understanding and to identify the key insights. This initial analysis was conducted following each interview to inform the planning of subsequent interviews. The interviews were continued until data saturation was achieved, which is indicated by the absence of new categories and the saturation of existing categories based on their characteristics and dimensions^30,31^. The interview data underwent conventional content analysis. In the first step each text was reviewed for immersion and acquiring insights and deep understanding around the phenomenon under study. In step 2 meaning units were determined based on the objectives and the study questions. In step3 important points were extracted as open codes, considering their clear and hidden meaning units. In step4 these codes were categorized under broader titles based on their similarities and differences, and in step 5 the data analysis continued until the themes were extracted^30,31^. In order to ensure the trustworthiness of the process, Guba and Lincoln criteria were used^32^. To enhance the credibility and reliability of the findings, various methods were utilized. These included a comprehensive examination of data sources such as semi-structured interviews, observation and field notes, as well as prolonged engagement with the data. Additionally, member checking and peer checking were employed to validate the extracted concepts and themes. Four participants and two peers were involved in this process, all of whom confirmed that the findings aligned with their own understandings and interpretations. The transferability of the study was ensured through a thorough description of the participants, interviews, and analysis. Furthermore, confirmability was achieved by accurately recording participant narratives and providing a detailed report of the study, facilitating the possibility of follow-up by other researchers. Finally, 1214 codes were which were categorized into twenty five subcategories, eleven categories, and three main themes, which were “moral sensitivity,” moral courage,” and “moral commitment” Fig. 1. During the study phase, there were an equal number of male and female participants, totaling 20 individuals, with an average age of 39.51 ± 2.51 years. Additionally, the majority of participants held a master's degree in nursing, possessed an average work experience of 12.98 + 1.15 years, and had a monthly income of approximately $550.

### Phase II. Psychometric properties

#### Questionnaire development

The assessment scale was created by generating 43 potential items from the qualitative data, which represented the main themes. Furthermore, seven additional items were included based on the findings from the literature review^14,21,22,24^, resulting in a total of 50 items. Subsequently, the research team evaluated the items and eliminated nine redundant ones, resulting in a final count of 41 items. These items were then categorized into three dimensions: "moral sensitivity" (consisting of 20 items), "moral courage" (comprising 10 items), and "moral commitment " (comprising 11 items).

#### Content validity

The content validity assessment involved consulting a panel of experts consisting of 15 professionals, including nurses, operating room nurses, doctors, and surgeons in the heart operating room. This expert panel evaluated the items regarding language, understanding, and suitability to the Iranian culture and context. Based on their evaluation, they suggested removing three items, leaving 38 questions for quantitative analysis of content validity using the content validity ratio (CVR) and content validity index (CVI)^33^. In order to accomplish this, the panel was given the instrument back, and they were requested to evaluate the items based on their relevance and importance to the study’s subject matter. According to the Lawshe table, the acceptable CVR was reported as 0.49^34,35^. However, five items with a CVR of 0.33 were removed from the study. The content validity index (CVI) was then evaluated for each remaining item. The revised instrument was given back to the panel, who were asked to rate each item’s relevance, simplicity, and clarity on a four-point Likert scale ranging from 1 to 4. The CVI was calculated for both individual items and the entire instrument. For this study, a CVI value greater than 0.8 was deemed acceptable^36^. However, three items had a score below this cut-off and were also deleted.

#### Face validity

The revised instrument with 30 items was then given to 30 healthcare professionals using the same inclusion criteria as for Phase 1. They were asked to assess each item regarding difficulties, relevance, grammar, vocabulary, and intelligibility. The participants declared that the items were simple, clear, and relevant to the study’s topic. In addition, an impact score was calculated in which participants evaluated each item using a five-point Likert scale ranging from one (very little (to five (very much), with a score > 1.5 considered acceptable^35^. The impact score for all items was higher than 1.5. Therefore, no further items were deleted.

#### Item analysis

A 30-item instrument was created based on the previous stage. Thirty eligible healthcare professionals used a five-point Likert scale (1 = very low, 2 = low, 3 = to some extent, 4 = high, 5 = very high) to rate themselves on the 30 items. The correlation coefficients between the items ranged from 0.3 to 0.7, and the total score across all items was calculated to be greater than 0.3^36^. All items met these criteria, and it was decided no further items were deleted. Finally, this scale includes ‘“moral sensitivity” (16 items), “moral commitment” (9 items), and moral courage” (5 items).

#### Participants and data collection

Three hundred healthcare professionals were recruited using a convenience sampling from six public centers providing care to patients in cardiac operating rooms affiliated with medical universities in Iran. The inclusion criteria were having at least 12 months of work experience in the cardiac operating room, being Iranian, having a good command of Farsi, and willingness to participate in the study. The participants’ socio-demographics were also collected. Data were analyzed using descriptive and inferential statistics via the SPSS software, v. 19 (SPSS Inc, Chicago, Illinois, USA). The mean participant’s age was 38.58 ± 2.78, ranging from 26 to 57 years. Most participating in this phase were men (57.33%), married (62.33%), had a bachelor’s degree (52.33%), had about 14.78 + 1.74 years of work experience and an average monthly income equal to 560 US dollars.

### Contrast validity (Exploratory factor analysis, convergent validity)

Construct validity helped ensure that the instrument measured what it intended to measure^35,36^. Exploratory factor analysis using the varimax rotation was used in this study. To achieve the most appropriate structure, eigenvalues higher than 1.0, factor loadings higher than 0.50, and the so-called ‘elbow criterion’ regarding the eigenvalues were considered^33,37^. The Kaiser–Meyer–Olkin (KMO) and Bartlett’s tests were performed to evaluate sample adequacy. For exploratory factor analysis, the KMO value had to be greater than 0.05. Pearson’s correlation coefficients were calculated between the developed instrument and the the moral comoetence scale for home care nurses scale for convergent validity.

### Confirmatory factor analysis

Confirmatory Factor Analysis was carried out utilizing AMOS 22 software, and several indices were employed to evaluate the model’s effectiveness. In order to ascertain the adequacy of the model, it was imperative to adhere to the following stringent criteria: goodness of fit index (GFI) exceeding 0.90, a root mean square error of approximation (RMSEA) below the acceptable threshold of 0.08, a Tucker Lewis Index (TLI) surpassing the minimum acceptable level of 0.90, and a comparative fit index (CFI) exceeding the requisite threshold of 0.90, as per established conventions^31,35^.

### Reliability

To ensure the validity of this instrument, both Cronbach’s alpha coefficient and test–retest reliability analysis were utilized. The internal consistency reliability was evaluated by calculating Cronbach’s alpha coefficient with a sample size of 300 participants. The acceptable Cronbach’s alpha coefficient was determined to be above 0.7. Fortest–retest reliability, the intra-class correlation (ICC) was calculated by collecting data from 70 participants at a two-week interval^37^.

### Ethical approval and consent to participate

Research Center the west of Iran provided ethics approval (IR.UMSHA.REC.1402.538). All methods were performed in accordance with the relevant guidelines and regulations, and all the research methods met the ethical guidelines described in the Declaration of Helsinki. Also, at the beginning of each interview, the researcher introduced herself, explained the study’s goals, and provided assurance that all information would remain confidential and that they could withdraw from the study at any time. They reassured participants that their decision to not participate or withdraw would not have any negative consequences for them. Lastly, the researchers obtained informed and written consent from all study participants.

## Results

### Contrast validity (Exploratory factor analysis, convergent validity)

Exploratory factor analysis using the varimax rotation identified three main factors, as shown in Table 1, which explained 73.04% of the observed variance together. The items’ factor loadings ranged from 0.608 to 0.923. The three included factors were ‘“moral sensitivity” (16 items), “moral commitment” (9 items), and moral courage” (5 items), which broadly confirmed the main themes identified in the qualitative data in Fig. 1. According to Pearson’s correlation, this instrument was significantly correlated with the moral comoetence scale for home care nurses, indicating moderate convergent validity (r = 0.65) (Table 2).

**Table 1:** Varimax factor loadings of the items of the Moral intelligence Scale for health care professional in the cardiac operating room.

Factors’ names	Item	Communality	Factor loading
Factor 1: Moral sensitivity	1-I respect the religious beliefs of patients	0.83	.923
	2-I pay attention to cultural sensitivities	.083	.915
	3-I respect the ethnic differences between patients	0.80	.892
	4-I treat patients with respect	0.78	.882
	5-I respect the differences in patients’ dialects and accents	0.76	.864
	6-I try to make patients understand my words correctly with different accents	0.75	.854
	7-I seek the assistance of colleagues who speak a dialect similar to the patients to communicate with them effectively	0.73	.848
	8-I address patients with appropriate words	0.721	.845
	9-With permission, I prepare the patients’ bodies for the procedure	0.71	.837
	10-I adhere to benevolence, honesty or fairness	0.70	.833
	11-I strive to ensure that proper care is taken for the same gender	0.69	.827
	12-I try to provide safe care	0.68	.774
	13-I try to keep patient information confidential	0.66	.722
	14-I respect patients’ right to choose and their authority	0.65	.718
	15-I Explain the procedures to the patients before the procedure	0. 61	.683
	16-I warmly welcome the patients in the operating room with a smile	0.60	.608
Factor 2: Moral commitment	17-I listen to patients’ concerns	0.80	.896
	18-I strive to reduce patients’ stress	0.78	.883
	19-I aim to gain patients’ trust	0.76	.869
	20-I explain medical information to patients in simple and understandable language	0.75	.863
	21-I work to improve my knowledge and performance	0.74	.852
	22-I try to manage my stress effectively	0.73	.846
	23-I guide novice nurses to appropriate performance	0.69	.737
	24-I guide new nurses on how to manage work pressure	0.67	.729
	25-I guide novice nurses on how to interact effectively with colleagues and patients	0.65	.721
Factor 3: Moral courage	26-I tell the truth even if it is to my disadvantage	0. 80	.891
	27-I accept responsibility for my mistakes	0.77	.861
	28-I report any mistakes made during procedures to the authorities	0.72	.834
	29-I accept constructive criticism	0.70	.751
	30-I strive not to allow any differences or discrimination among patients	0.69	.748

**Table 2:** Convergent validity of the Moral intelligence Scale for health care professionals in the cardiac operating room with the moral comoetence scale for home care nurses scale.

Scale	The moral comoetence scale for home care Nurses scale	
This instrument	Pearson r	0.65
	*P* value	< .03*

*Correlation is significant.

### Confirmatory factor analysis

**Figure 2:**
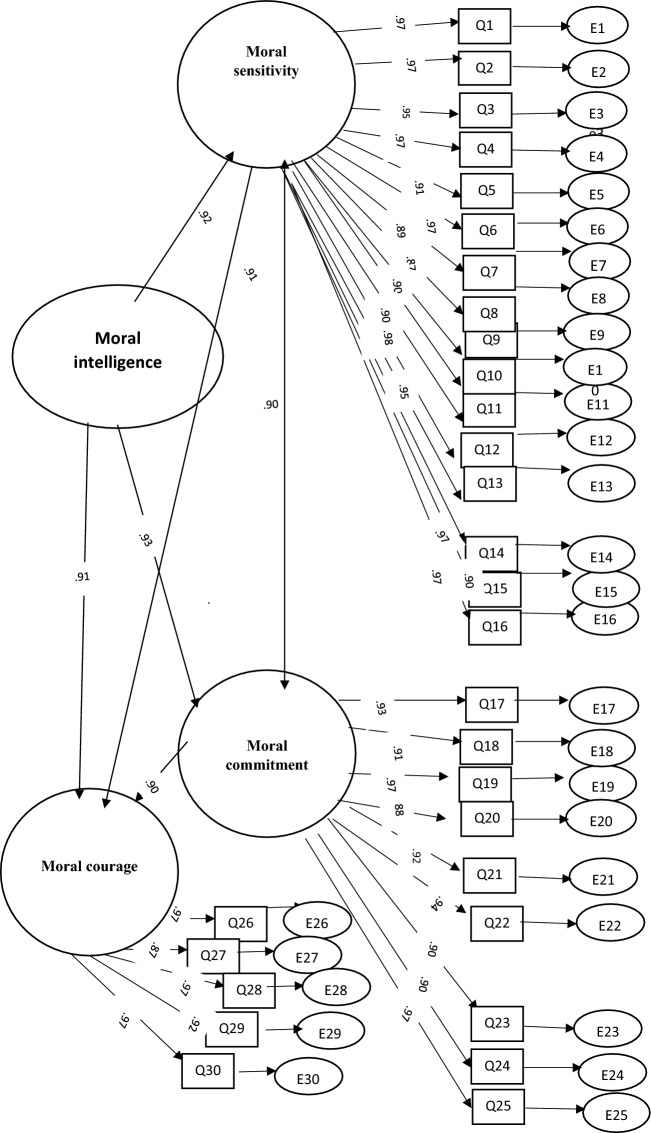
Model fit the moral intelligence scale.

The result of confirmatory factor analysis indicated one model with three factors ‘“moral sensitivity” (16 items), “moral commitment” (9 items), and “moral courage” (5 items). ‘moral sensitivity’ showed a 0.92 correlation, ‘moral commitment’ showed 0.93, and “moral courage” showed a 0.91 correlation with a total score of moral intelligence. Also, there was a correlation between two factors, moral sensitivity and moral commitment 0.90, and between moral sensitivity and moral courage 0. 91. Also, there was a correlation between moral commitment and moral courage 0.90. The chi-square of 532.13 (df = 85, *P* = 0.032) showed good fitness. In addition, the GFI in the current study was 0.94, which showed a good fitting with the uni-dimensional model of the PTES construct. Further indices were tested in this model: RMSEA = 0.04, CFI = 0.96, NFI = 0.94, and TLI = 0.95. All of the reported indices indicated that the extracted model fit the moral intelligence scale well (Fig. 2).

### Reliability

The reliability of the questionnaire was assessed using cronbach’s alpha coefficient and test–retest reliability. The cronbach’s alpha coefficient of internal consistency across the 30-item instrument was 0.94, and for the three subscales of ‘“moral sensitivity,” “moral commitment” and “moral courage” were 0.94, 0.93, and 0.95, respectively. The test–retest reliability of the questionnaire was calculated by inviting 70 healthcare professionals to complete the questionnaire again after a two-week interval. The test–retest showed no statistically significant difference between pre-and post-test scores (*p* = 0.51). The correlations between the scores on the moral sensitivity of the questionnaire between test–retest were 0.91, and the correlations between the scores on moral commitment of the questionnaire between test–retest were 0.90, and the correlations between the scores on the moral courage of the questionnaire between the test–retest were 0.92. Finally, the correlation coefficient of the test–retest is 0.91 (Table 3).

**Table 3:** Mean (standard deviation) and intraclass correlation coefficient (ICC) values for the domains of the Moral intelligence Scale for health care professional in the cardiac operating room.

Factor	Dimensions	Mean ± SD	ICC	Confidence interval
1	Moral sensitivity	65.17 (3.44)	0.91	0.74–0.90
2	Moral commitment	38.14 (3.41)	0.90	0.85–0.96
3	Moral courage	20.125 (2.18)	0.92	0.82–0.94
Moral intelligence Scale (total)		135.67 (3.21)	0.91	0.86–0.932

Finally, an instrument with 30 items was developed that includes ‘“moral sensitivity” (16 items), “moral commitment” (9 items), and moral courage” (5 items). All items were scored based on a five-point Likert scale (1 = very low to 5 = very high); the scale was designed to be completed within 20 min. The total score range is from 30 to 150. Higher scores indicate more moral intelligence. Also, the range of scores shows 30–70 (low moral intelligence), 71–110 (moderate moral intelligence), and 111–150 (high moral intelligence). The moral intelligence scale of health- care professionals in the cardiac operating room is shown in Table 4

**Table 4:** Moral intelligence Scale of Health-care Professionals in the Cardiac Operating Room.

Dimensions	Questions	Very low	Low	To some extent	High	Very high
Moral sensitivity	1-I respect the religious beliefs of patients					
	2-I pay attention to cultural sensitivities					
	3-I respect the ethnic differences between patients					
	4-I treat patients with respect					
	5-I respect the differences in patients’ dialects and accents					
	6-I try to make patients understand my words correctly with different accents					
	7-I seek the assistance of colleagues who speak a dialect similar to the patients to communicate with them effectively					
	8-I address patients with appropriate words					
	9-With permission, I prepare the patients’ bodies for the procedure					
	10-I adhere to benevolence, honesty, or fairness					
	11-I strive to ensure that proper care is taken for the same gender					
	12-I try to provide safe care					
	13-I try to keep patient information confidential					
	14-I respect patients’ right to choose and their authority					
	15-I Explain the procedures to the patients before the procedure					
	16-I warmly welcome the patients in the operating room with a smile					
Moral commitment	17-I listen to patients’ concerns					
	18-I strive to reduce patients’ stress					
	19-I aim to gain patients’ trust					
	20-I explain medical information to patients in simple and understandable language					
	21-I work to improve my knowledge and performance					
	22-I try to manage my stress effectively					
	23-I guide novice nurses to appropriate performance					
	24-I guide new nurses on how to manage work pressure					
	25-I guide novice nurses on how to interact effectively with colleagues and patients					
Moral courage	26-I tell the truth even if it is to my disadvantage					
	27-I accept responsibility for my mistakes					
	28-I report any mistakes made during procedures to the authorities					
	29-I accept constructive criticism					
	30-I strive not to allow any differences or discrimination among patients					

## Discussion

The moral intelligence of healthcare professionals in the operating room has been defined as a combination of moral sensitivity, moral commitment, and moral courage for the provision of quality care that respects the principles of medical ethics. Then, based on the inferred conceptual framework, the moral intelligence scale of healthcare professionals in the cardiac operating room was designed and psychometrically evaluated. This scale was designed in three dimensions with 41 items. In measuring the content validity 11 items were removed, and 30 items were selected for face validity. The scale consists of 30 items across three dimensions: moral sensitivity (16 items), moral commitment (9 items), and moral courage (5 items). All 30 items had an impact score greater than 1.5 during the face validity assessment, and no items were removed. Exploratory and confirmatory factor analysis confirmed the structure of this scale without any item removal or replacement. The scale’s reliability was acceptable, as reported by Cronbach’s alpha and test–retest reliability. These findings indicate the appropriateness of this scale for measuring the moral intelligence of healthcare professionals in the operating room in the Iranian community.

It should be noted that appropriate and accessible scales for measuring moral intelligence as an important aspect of the professional competency of healthcare professionals are inadequate. In this regard, three scales have been used in studies to assess the moral competence and intelligence of healthcare professionals, which are referred to for a comprehensive discussion.

Asahara and colleagues developed a moral comoetence assessment scale for nurses in home care in Japan in 2013. This scale consists of 45 items in 5 dimensions: moral sensitivity, judgment, motivation, personality, and decision-making. The scale has appropriate face and content validity. Additionally, exploratory validity reports item factor loading between 0.41 and 0.93. Furthermore, it has acceptable confirmatory validity, and reliability for the 5 dimensions with reports of 0.78 to 0.93, which is suitable and acceptable, so it is in line with the current study^26^. However, it should be noted that this scale was designed to measure the moral comoetence of nurses in home care and in a cultural context different from the Iranian community. Moreover, the needs of patients in the operating room, especially in the cardiac operating room, are different from those requiring care at home. Therefore, although some items for assessing the moral intelligence of health caregivers may be similar between the two studies, the moral intelligence of health caregivers in the operating room is somewhat different from those of in-home care due to the different care environments and patient needs. Hence, there is a need for a specific scale to be designed.

One of the other most commonly used scales for measuring the moral competence and intelligence of healthcare professionals is the extended version of the moral competence test. This scale evaluates individuals’ attitudes, decision-making, and moral performance by presenting three moral dilemma questions. The scale has exhibited satisfactory validity and reliability^24,38^. The purpose of this scale is not to evaluate the moral intelligence of caregivers. Additionally, its use for assessing moral attitudes and judgment is more complex and problematic than other scales. Consequently, it is not frequently utilized in the medical field for evaluating moral intelligence. Thus, there is a necessity for a more specialized scale.

The competency inventory for registered nurses (CIRN) is one of the most important scales used to measure the moral competence and intelligence of healthcare professionals. This scale assesses professional competence across 55 items in 7 dimensions, including critical thinking, research inclination, clinical care, leadership, interpersonal communication, legal and moral performance, and professional development and education. One dimension focuses solely on moral performance, consisting of 8 items, some similar to the items in the scale designed for this study. The scale has appropriate and acceptable CVR, CVI, structural validity, and reliability, making it compatible with this study^39,40^. However, although this scale has appropriate validity and reliability, it was not exclusively designed to measure the moral intelligence of healthcare professionals. On the other hand, moral intelligence is a concept that depends on culture, atmosphere, and environmental factors. The importance of designing a specific scale to measure moral intelligence in healthcare professionals is highlighted by the high-stress and tension-filled environment of the operating room, especially the cardiac operating room.

## Limitation

Firstly, the participants were restricted to healthcare professionals exclusively working in the cardiac operating room at public care centers. To broaden the scope of our findings, it would be advantageous to involve healthcare professionals from private care centers as well. It is also suggested that in future studies, the psychometric evaluation of this scale should be done in other surgical and non-surgical departments. Another limitation of this study was the homogeneity of the sample, single-country setting, and cross-sectional design. So, testing of this instrument’s psychometric properties is suggested, involving a larger population of healthcare professionals in different cultures. Another limitation of this study was the lack of sensitivity to the external validity of the scale in this study due to the study being conducted at one point in time. Based on this, it is suggested to psychometrically evaluate this instrument and accurately evaluate its responsiveness, sensitivity, and external validity in multiplicity studies and in check the length of time.

## Conclusion

The participants in this study defined the moral intelligence of healthcare professionals in the cardiac operating room as a combination of moral sensitivity, moral commitment, and moral courage to esnure the delivery of quality care that respects the principles of medical ethics. Based on the inferred conceptual framework, the moral intelligence scale of healthcare professionals in the cardiac operating room was designed with 30 items in three dimensions: appropriate face, content, exploratory, and confirmatory validity and reliability. Hence, this tool can be employed to evaluate the moral intelligence of healthcare practitioners and identify the necessity for educational interventions.

## Data Availability

The data supporting this study’s findings are available from the corresponding author upon reasonable request.
